# Antagonistic Roles for GcvA and GcvB in *hdeAB* Expression in *Escherichia coli*

**DOI:** 10.5402/2012/697308

**Published:** 2012-05-16

**Authors:** Lorraine T. Stauffer, George V. Stauffer

**Affiliations:** Department of Microbiology, University of Iowa, Iowa City, IA 52242, USA

## Abstract

In *E. coli*, the periplasmic proteins HdeA and HdeB have chaperone-like functions, suppressing aggregation of periplasmic proteins under acidic conditions. A microarray analysis of RNA isolated from an *E. coli* wild type and a ΔgcvB strain grown to mid-log phase in Luria-Bertani broth indicated the *hdeAB* operon, encoding the HdeA and HdeB proteins, is regulated by the sRNA GcvB. We wanted to verify that GcvB and its coregulator Hfq play a role in regulation of the *hdeAB* operon. In this study, we show that GcvB positively regulates *hdeA*::*lacZ* and *hdeB*::*lacZ* translational fusions in cells grown in Luria-Bertani broth and in glucose minimal media + glycine. Activation also requires the Hfq protein. Although many sRNAs dependent on Hfq regulate by an antisense mechanism, GcvB regulates *hdeAB* either directly or indirectly at the level of transcription. GcvA, the activator of *gcvB*, negatively regulates *hdeAB* at the level of transcription. Although expression of *gcvB* is dependent on GcvA, activation of *hdeAB* by GcvB occurs independently of GcvA's ability to repress the operon. Cell survival and growth at low pH are consistent with GcvA negatively regulating and GcvB positively regulating the *hdeAB* operon.

## 1. Introduction

 Acid resistance is important for the ability of enteric bacteria to survive the low pH environment encountered in the gastrointestinal tract of mammalian hosts and other natural environments [1]. Enteric bacteria have five systems of acid resistance [2–7]. The first system, AR1, is least understood. When cells are grown in LB at pH 5 to stationary phase, they survive dilution into minimal medium at pH 2.5, which kills cells grown at pH 8. The stationary phase sigma factor RpoS and cyclic-AMP receptor protein are required to develop acid tolerance [2, 6]. The other four systems, AR2 AR3, AR4, and AR5, are decarboxylate/antiporter-dependent acid resistance systems that require glutamate, arginine, lysine, and ornithine, respectively [2, 4–10]. Additional acid protection comes from the periplasmic proteins HdeA and HdeB that have chaperone-like functions, suppressing aggregation of periplasmic proteins under extreme acidic conditions [11–13]. Both *hdeA* and *hdeB* mutants show reduced viability upon acid stress and HdeA/HdeB expressing plasmids restore viability close to wild type, suggesting both proteins are necessary for protection of the bacterial periplasm against acid stress [14]. Regulation of the *hdeAB* operon is complex. The *hdeAB* operon in *E*. *coli* is acid inducible and regulation involves GadE, RpoD, RpoS, H-NS, MarA, and several other regulators [6, 7, 15–18].

 The *E*. *coli gcvB* gene encodes a sRNA of 206 nucleotides [19]. Expression of *gcvB* is activated by the GcvA protein when cellular glycine is high and repressed by GcvA and GcvR when glycine is limiting [19]. In both *E*. *coli* and *Salmonella enterica *serovar Typhimurium, GcvB regulates genes involved in the transport of small peptides and polar and branched amino acids [19–24]. Recently, it was shown GcvB enhances the ability of *E*. *coli* to survive low pH by upregulating RpoS [25]. In addition, microarray data suggested the *hdeAB* operon is positively regulated by GcvB [22]. Results from this study establish a role for GcvA in repressing the *hdeAB* operon and GcvB in activating the operon. Hfq, an RNA chaperone required for GcvB regulation of known target genes [20, 22, 23, 26], is also required for activation. However, the results suggest GcvB and Hfq do not function as an antisense RNA system to upregulate *hdeAB* translation, but act at the level of transcription. The results also suggest GcvA, the activator for *gcvB*, negatively regulates *hdeAB* at the level of transcription.

## 2. Materials and Methods

### 2.1. Bacterial Strains, Plasmids, and Phage

The *E*. *coli* strains, plasmids, and phage used are listed in Table 1 or described in the text. 

### 2.2. Construction of Recombinant Phages and Plasmids

**Figure 1:**
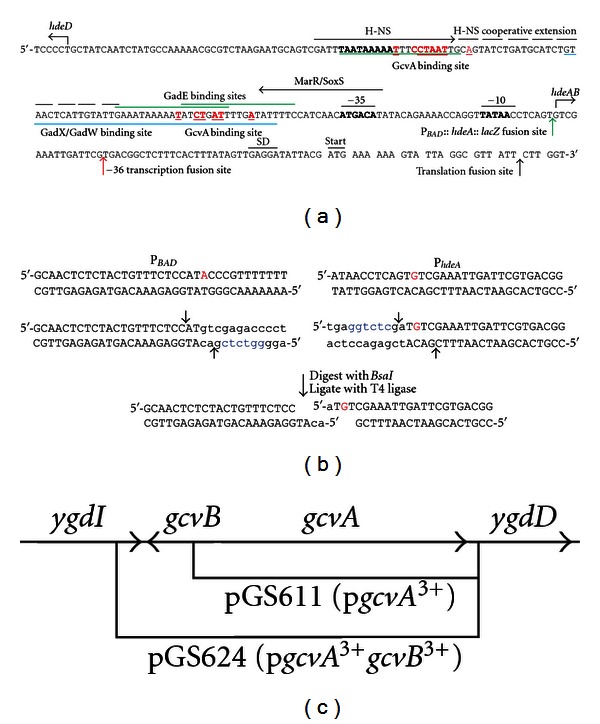
(a) The *hdeAB* control region. The *hdeAB* promoter −35, −10 and transcription start site and the SD sequence and translation start site are indicated above the sequence [31]. The transcription start site for the divergently transcribed *hdeD* gene is also shown [32]. Binding sites for H-NS [16] and MarR/SoxS [15] are indicated above the sequence with arrows. The binding site for GadX/W [33] is below the sequence in blue and for GadE above the sequence in green [18, 34]. In addition, there are putative binding sites for the transcriptional regulators Lrp and TorR (not shown) [34]. The consensus GcvA binding site is T-N_11_-A containing a 5′-CTAAT-3′ sequence [35]. Two putative GcvA binding sites are indicated in red. The fusion sites for the *λhdeA*::*lacZ* translational fusion, the *λ* *hdeA^−36^*::*lacZ* transcriptional fusion, and the *λ*P*_BAD_*::*hdeA*::*lacZ* fusion (see below) are indicated with black, red, and green arrows, respectively. (b) Construction of a *λ*P*_BAD_*::*hdeA*::*lacZ* promoter fusion. The WT P*_BAD_* and P*_hdeA_* promoters are shown in the top line. The transcription start sites are in red [31, 36]. Small case letters show bases added during PCR amplification of the P*_BAD_* and P*_hdeA_* promoters. The P*_BAD_* promoter was amplified with an upstream primer containing an *Eco*RI site at bp −272 relative to the transcription start site (not shown) and a downstream primer with a *Bsa*I site (blue). The P*_hdeA_* promoter was amplified with an upstream primer containing a *Bsa*I site (blue) and a downstream primer containing a *Sma*I site at codon 10 in the *hdeA* gene (not shown). The arrows indicate cut sites for *Bsa*I. The amplified products were cut with *Bsa*I, mixed, and ligated, generating a fusion of the P*_BAD_* promoter with the +1G residue of the P*_hdeA_* promoter. The fragment was then digested with *Eco*RI + *Sma*I and ligated into the *Eco*RI-*Sma*I sites of plasmid pMC1403, and subsequently subcloned into *λ*gt2 as described [19]. (c) The *gcvA gcvB* region of the *E*. *coli* chromosome. The regions amplified by PCR and cloned into pACYC184 to generate pGS611 (p*gcvA* ^3+^) and pGS624 (p*gcvA* ^3+^ *gcvB* ^3+^) are indicated with bars. See Section 2.2 for details.

 The *λhdeA*::*lacZ* translational fusion was constructed by PCR synthesis of a DNA fragment using an upstream primer with an *Eco*RI site that hybridized to DNA beginning 223 bps upstream of the *hdeA* transcription start site and a downstream primer with a *Sma*I site that hybridized to DNA beginning at codon 7 within the *hdeA* gene. The PCR amplified DNA fragment was digested with *Eco*RI + *Sma*I and the 303 bp *Eco*RI-*Sma*I fragment ligated into the *Eco*RI-*Sma*I sites of plasmid pMC1403 [37], fusing the first 7 codons of the *hdeA* gene in frame with the 8th codon of the *lacZYA* genes in pMC1403 (Figure 1(a)). The cloned sequence was verified by DNA sequence analysis at the DNA Core Facility of the University of Iowa. The plasmid was designated p*hdeA*::*lacZ*. A 5,574 bp *Eco*RI-*Mfe*I fragment from p*hdeA*::*lacZ* carrying the *hdeA*::*lacZYA* fusion was then ligated into the *Eco*RI site of phage *λ*gt2 [30], generating *λhdeA*::*lacZ*. A *λhdeB*::*lacZ* fusion was constructed using the same upstream primer and a downstream primer with a *Sma*I site that hybridized to DNA beginning at codon 9 within the *hdeB* gene. The 757 bp *Eco*RI-*Sma*I fragment was then used as described above, generating plasmid p*hdeB*::*lacZ* and phage *λhdeB*::*lacZ* (not shown). A *λhdeA*::*lacZ *transcriptional fusion was constructed using the same upstream primer and a downstream primer with a *Hind*III site and that hybridized to DNA at bp −36 relative to the *hdeA* translation start site (Figure 1(a)). Following digestion with *Eco*RI and *Hind*III, the DNA fragment was ligated into the *Eco*RI and *Hind*III sites of plasmid p*gcvB- lacZ* ^+50^ [19], replacing the *gcvB* fragment with the *hdeA *fragment, generating plasmid p*hdeA* ^−36^::*lacZ*. The cloned sequence was verified by DNA sequence analysis. A 5,538 bp *Eco*RI-*Mfe*I fragment from p*hdeA* ^−36^::*lacZ* carrying the *hdeA* ^−36^::*lacZYA* fusion was then ligated into the *Eco*RI site of phage *λ*gt2 [30], generating *λhdeA* ^−36^::*lacZ*. The 3 fusion phages were used to lysogenize *E*. *coli* host strains as described [38]. Each lysogen was tested to ensure it carried a single-copy of the *λ* chromosome by infection with *λc*I90*c*17 [39]. All lysogens were grown at 30°C since all fusion phage carry the *λc*I857 mutation, resulting in a temperature-sensitive *λc*I repressor [30]. The *λ*P*_BAD_*::*hdeA*::*lacZ* fusion, where *hdeA* transcription is under control of the P*_BAD_* promoter, was constructed as described in Figure 1(b).

 Plasmid pGS611 (p*gcvA* ^3+^), carrying the *E*. *coli gcvA* gene on a 1,155 bp *Eco*RI fragment, was constructed as follows. In a PCR reaction, an upstream primer was used containing an *Eco*RI site and that hybridized to a region beginning 121 bp upstream of the *gcvA* transcription start site and a downstream primer containing an *Eco*RI site and hybridized to a region beginning 44 bp downstream of the *gcvA* translation stop codon. The *Eco*RI sites were added as parts of the primers. The PCR-generated fragment was digested with *Eco*RI and cloned into the *Eco*RI site in plasmid pACYC184 [40] and verified by DNA sequence analysis (Figure 1(c)). Plasmid pGS624 (p*gcvA* ^3+^ *gcvB* ^3+^), carrying both the *gcvA* and *gcvB* genes, was constructed in the same way except the upstream primer hybridized to DNA 51 bps after the *gcvB* transcription terminator and the downstream primer hybridized to DNA 44 bps after the *gcvA* translation stop codon, generating a 1,347 bp *Eco*RI fragment (Figure 1(c)).

### 2.3. Media

 The complex medium used was LB [41]. Agar was added at 1.5% (w/v) to make solid media. The minimal medium used was the salts of Vogel and Bonner [42] supplemented with 0.4% (w/v) glucose (GM). Ampicillin was added at 50 and 150 *μ*g mL^−1^ when strains carried single-copy and multicopy plasmids, respectively. Other supplements were added at the following concentrations (*μ*g mL^−1^): phenylalanine, 50; glycine, 300; thiamine, 1; TC, 10; CM, 20; X-gal, 40.

### 2.4. DNA Manipulation

 Plasmid DNA was isolated using a QIAprep Spin Miniprep Kit (Qiagen, Santa Clara, CA). Vent DNA polymerase and restriction enzymes were from New England Biolabs, Inc. (Beverly, MA). T4 DNA ligase was from Roche Diagnostics (Indianapolis, IN). Reactions were as described by the manufacturers.

### 2.5. Enzyme Assay

*β*-galactosidase assays were performed on mid-log phase cells (OD_600_ ~ 0.5) using the chloroform/SDS lysis procedure [41]. Results are the averages of two or more assays with each sample done in triplicate.

### 2.6. Acid Sensitivity Assay

**Figure 2:**
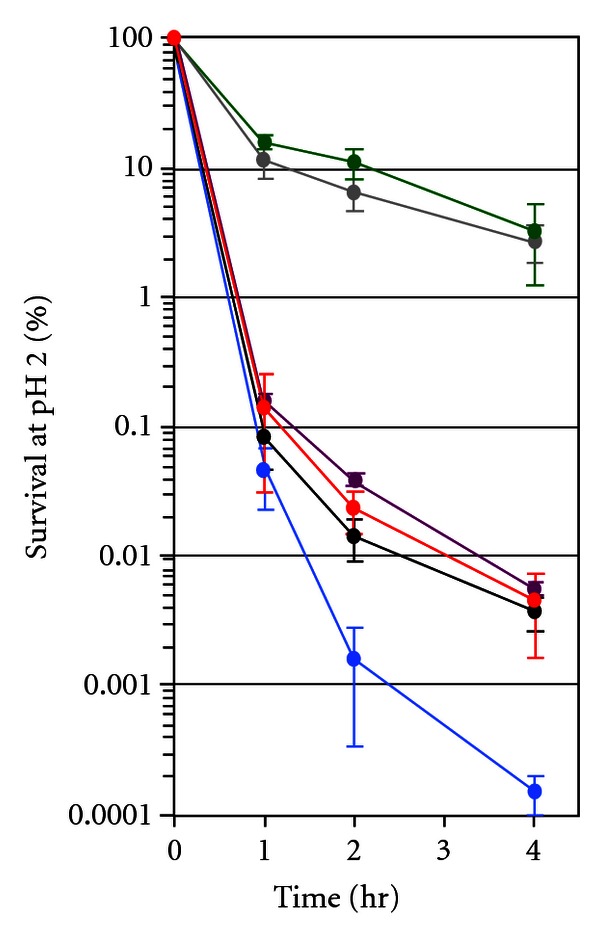
Percent survival of *E*. *coli* strains plotted against time at pH 2.0. Black, WT; gray, Δ*gcvAB*; green, Δ*gcvAB*[p*gcvB* ^2+^]; blue, WT[p*gcvA* ^3+^ *gcvB* ^3+^]; purple, Δ*gcvAB*[p*gcvA* ^3+^ *gcvB* ^3+^]; red, Δ*gcvAB*[p*gcvA* ^3+^]. See Section 2.6 for details.

 WT, an isogenic Δ*gcvAB *strain and the two strains transformed with either plasmid p*gcvB* ^2+^ (constitutively produces GcvB), p*gcvA* ^3+^ or p*gcvA* ^3+^ *gcvB* ^3+^ were grown for 24 hr at 30°C in LB and then tested for acid resistance by dilution into LB at pH 2.0. Samples of 0.2 mL were taken at 0, 1, 2, and 4 hr and diluted in 2 mL of LB at pH 7. The final pH of the diluted cultures was ~7.0. Cell viability was determined by plate counts. Percent survival is the titer of colony forming units of acid-tested cells compared to the zero-time point (Figure 2).

### 2.7. Transductions

The *gcvB* gene is linked to the *argA* gene and *hfq* is linked to the *cycA* gene, with predicted phage P1 cotransduction frequencies of ~78% and ~67%, respectively. P1*clr* phage prepared on GS854 (*argA81*::Tn*10*) was used to transduce Δ*gcvB*::ΩCM^R^ *λhdeA*::*lacZ* to TC^R^ and transductants scored on CM versus TC plates. A TC^R^ CM^S^ transductant was purified. P1*clr* prepared on GS776 (*cycA*::Tn*10*) was used to transduce Δ*hfq-1*::ΩCM^R^ *λhdeA*::*lacZ* to TC^R^ resistance and transductants scored on CM versus TC plates. A TC^R^ CM^S^ transductant was purified.

## 3. Results and Discussion

### 3.1. GcvA/GcvB Role in Acid Sensitivity

 Microarray data suggested the *hdeA *and *hdeB* mRNAs are 1.9- and 2.7-fold higher in WT than a Δ*gcvB* strain grown in LB, respectively [22]. These genes were not reported to be regulated by GcvB in that study because they fell below the 3-fold cut-off level used for GcvB-regulated genes. Since HdeA and HdeB are necessary for protection of the bacterial periplasm against acid stress [14, 17], we tested if GcvB plays a role in cellular acid resistance. WT and an isogenic Δ*gcvAB *strain were grown for 24 hr at 30°C in LB and tested for acid resistance by dilution into LB at pH 2.0 [43]. The WT was killed significantly more readily at pH 2 than the Δ*gcvAB* strain (Figure 2, compare black and gray lines). However, when the Δ*gcvAB* strain was transformed with p*gcvB* ^2+^ that constitutively expresses GcvB [19], we did not see complementation that restored acid sensitivity (Figure 2, green line). When transformed with the multi-copy plasmid p*gcvA* ^3+^ *gcvB* ^3+^, both the WT and the Δ*gcvAB* transformants were more acid sensitive (Figure 2, compare the black and blue lines and the gray and purple lines). Plasmid p*gcvA* ^3+^, which carries only the *gcvA* gene, also complemented the Δ*gcvAB* mutation, increasing acid sensitivity (Figure 2, compare the gray and red lines). The results suggest it is the absence of GcvA that is responsible for increased acid resistance in the Δ*gcvAB* strain. It was reported previously that GcvB plays a positive role in acid resistance [25]. Our failure to observe a significant effect on acid resistance is possibly due to the assay conditions. We tested for acid resistance after 24 hours of growth in LB, whereas in the earlier study acid resistance was tested after 5 hr of growth in LB [25]. Although the precise stage of growth was not stated in the earlier study, it is possible cells were still in log phase. In *E*. *coli* and *Salmonella* grown in LB, GcvB was only detected through early stationary phase, with the highest levels observed at the mid-exponential phase [20, 26]. Thus, GcvB regulation of target genes involved in acid resistance is likely during log phase and if GcvB plays a role in stationary phase, it is its absence that is important for allowing an appropriate regulatory response.

### 3.2. Effects of GcvB on **λ*hdeA*::*lacZ* Expression in LB Grown Cells

**Figure 3:**
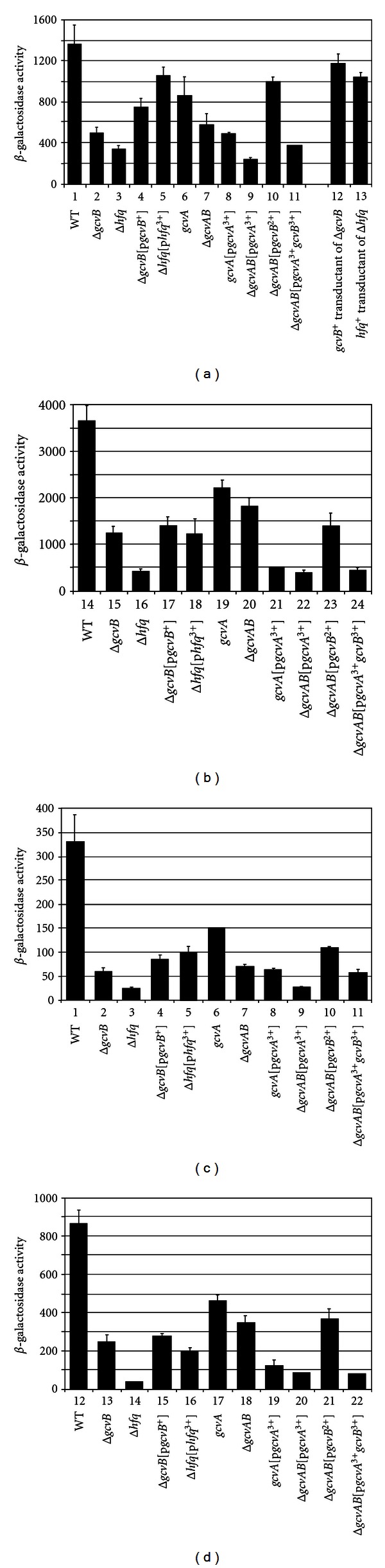
Effect of GcvB, Hfq, and GcvA on *λhdeA*::*lacZ* and *λhdeB*::*lacZ* expression. *λhdeA*::*lacZ* lysogens were grown in (a) LB or (b) GM + glycine to mid-log phase and assayed for *β*-galactosidase activity. *λhdeB*::*lacZ* lysogens were grown in (c) LB or (d) GM + glycine to mid-log phase and assayed for *β*-galactosidase activity.

 Although GcvB had no effect in the acid sensitivity assay, we made and tested expression of *λhdeA*::*lacZ* and *λhdeB*::*lacZ *translational fusions. Expression of the *hdeA*::*lacZ* fusion was 2.7- and 4-fold higher in WT grown in LB compared to Δ*gcvB* and Δ*hfq* strains (Figure 3(a), compare lanes 1, 2 and 3). Activation was partially restored in the Δ*gcvB*[p*gcvB^+^*] and Δ*hfq*[p*hfq* ^3+^] complemented strains (Figure 3(a), compare lanes 2 and 4 and lanes 3 and 5). It is unknown why the plasmids fail to fully complement the Δ*gcvB* and Δ*hfq* mutations. Nevertheless, the results agree with microarray data and suggest GcvB and Hfq positively regulate *hdeA*::*lacZ*. 

### 3.3. Reduced *hdeA*::*lacZ* Expression in ΔgcvB and Δhfq Strains Is due to the Absence of GcvB and Hfq

 Due to the failure of p*gcvB* ^+^ and p*hfq* ^3+^ to fully complement the *gcvB* and *hfq* mutations (Figure 3), we wanted to verify the reduced levels of *hdeA*-*lacZ* expression are due to the absence of GcvB and Hfq. We transduced the Δ*gcvB* and Δ*hfq* lysogens with WT alleles using linked Tn*10* markers. The *gcvB* ^+^ and *hfq* ^+^ transductants showed about the same levels of expression as the WT lysogen (Figure 3(a), compare lanes 1, 12 and 13). Thus, despite the failure of p*gcvB* ^+^ and p*hfq* ^3+^ to fully complement, the results support the reduced levels of expression are due to the absence of GcvB and Hfq.

### 3.4. Effects of GcvA on **λ*hdeA*::*lacZ* Expression in LB Grown Cells

 The acid sensitivity assay showed GcvA plays a role in acid resistance (Figure 2). In addition, putative GcvA binding sites can be identified in the *hdeA* promoter region (Figure 1(a)). Thus, we tested the effects of a spontaneous *gcvA* mutation in strain GS1198 (which is phenotypically GcvB^−^ [19]), on *hdeA*::*lacZ* expression. Expression of *hdeA*::*lacZ* was ~1.5-fold higher in WT than in the *gcvA* mutant (Figure 3, lanes 1 and 6). However, expression was 2-fold higher in the *gcvA* lysogen than in the Δ*gcvB* lysogen (Figure 3(a), compare lanes 2 and 6). The results could be explained if GcvA, in addition to activating expression of *gcvB*, which encodes a positive regulator for *hdeA*, also has a negative role to keep HdeAB levels low. The intermediate level of expression would result from the absence of GcvB to upregulate the *hdeA*::*lacZ* fusion and the absence of GcvA to negatively regulate the fusion. To test this hypothesis, we transformed the *gcvA* mutant with multi-copy p*gcvA* ^3+^. In the *gcvA*[p*gcvA* ^3+^] lysogen, one would expect high GcvA levels, but GcvB would also be produced. Expression of *hdeA*::*lacZ* was 2.5-fold higher in WT than in the *gcvA*[p*gcvA* ^3+^] transformant (Figure 3(a), compare lanes 1 and 8). In addition, *hdeA*::*lacZ* expression was reduced 2-fold compared to the nontransformed *gcvA* strain (Figure 3(a), lanes 6 and 8). We then transformed the Δ*gcvAB* lysogen with p*gcvA* ^3+^. In the Δ*gcvAB*[p*gcvA* ^3+^] lysogen, there would be high GcvA levels and no GcvB, and repression of *hdeA*::*lacZ* should be greatest. In the Δ*gcvAB*[p*gcvA* ^3+^] lysogen there was a 5.8-fold reduction of *hdeA*-*lacZ* expression compared to WT and a 2.4-fold reduction compared to the Δ*gcvAB* lysogen (Figure 3(a), compare lanes 1, 7 and 9). The results support a role for GcvA in negatively regulating *hdeA*::*lacZ* expression.

### 3.5. GcvB Positively Regulates *hdeA*::*lacZ* Independent of GcvA

If GcvA plays a negative role in *hdeA*::*lacZ* expression, GcvB could function to prevent the GcvA effect. Alternatively, GcvB could function independent of GcvA to activate *hdeA*::*lacZ*. To test these two possibilities, we transformed the Δ*gcvAB* *λhdeA*::*lacZ* lysogen with p*gcvB* ^2+^, which makes GcvB constitutively [19]. If GcvB's role is to block GcvA's ability to repress *hdeA*::*lacZ* expression, we hypothesized there would be no effect of GcvB in a Δ*gcvAB* *λhdeA*::*lacZ* lysogen without GcvA. Alternatively, if GcvB positively regulates *hdeA*::*lacZ*, we hypothesized expression of *gcvB* would increase *hdeA*::*lacZ* expression. In the Δ*gcvAB*[p*gcvB* ^2+^] lysogen, *hdeA*::*lacZ* expression increased 1.7-fold compared to the non-transformed lysogen, almost to the WT level (Figure 3(a), compare lanes 1, 7 and 10). We also transformed the Δ*gcvAB* *λhdeA*::*lacZ* lysogen with p*gcvA* ^3+^ *gcvB* ^3+^, which overproduces both GcvA and GcvB. Repression of *hdeA*::*lacZ* was restored, but not as low as in the p*gcvA* ^3+^ transformant (Figure 3(a), compare lanes 9 and 11). It is likely that the high GcvB levels partially negate the effect of high GcvA levels. The results suggest GcvB plays a role in activating *hdeA*::*lacZ* independent of GcvA.

### 3.6. Effect of GcvA, GcvB, and Hfq on *hdeA*::*lacZ* Expression in GM + Glycine

In *E*. *coli*, GcvB represses *dppA*::*lacZ*, *oppA*::*phoA*, *cycA*::*lacZ,* and *sstT*::*lacZ* fusions when cells are grown in LB, but does not significantly repress these fusions when grown in GM + glycine [19, 22, 23]. However, *gcvB* is differentially regulated over a 25-fold range in GM supplemented with inosine versus glycine [19]. We hypothesize some genes respond to GcvB levels in GM media. Microarray data suggested the *hdeA *mRNA is 1.6-fold higher in WT than a Δ*gcvB* strain grown in GM + glycine [22]. In GM + glycine, *hdeA*::*lacZ* expression was significantly higher than for cells grown in LB (Figure 3, compare a and b). In addition, although there are small differences in fold regulation for individual strains, there was a similar regulatory pattern in GM + glycine as observed in LB. The results suggest GcvB positively regulates *hdeA* in LB and GM + glycine. The results are important since they confirm GcvB does regulate in GM + glycine. In addition, acid resistance mechanisms are most active in the stationary phase in rich media [6, 7, 44, 45]. Our results suggest GcvA and GcvB could play important roles in acid resistance during the log phase of growth in both rich and minimal medium.

### 3.7. Effect of GcvA, GcvB, and Hfq on *hdeB*::*lacZ* Expression

 The *hdeB* gene is the second gene in the *hdeAB* operon. We tested if *hdeB* is regulated in a manner similar to the *hdeA* gene. There were small differences in the levels of *hdeB*::*lacZ* expression in response to GcvB, GcvA, and Hfq compared to *hdeA*::*lacZ* in both LB and GM + glycine (compare Figures 3(a) and 3(b) with Figures 3(c) and 3(d)). Qualitatively, however, the *λhdeB*::*lacZ* fusion showed essentially an identical pattern of expression compared to the *hdeA*::*lacZ* fusion, suggesting both genes of the operon are regulated in a similar manner by GcvA, GcvB, and Hfq.

 It is worth noting that Δ*hfq* lysogens consistently showed lower levels of *hdeA*::*lacZ* and *hdeB*::*lacZ* expression than Δ*gcvB* lysogens in both LB and GM + glycine (Figure 3). Two other sRNAs, DsrA, and GadY, are known to play roles in regulation of acid-resistance genes [46, 47]. Since both sRNAs require Hfq, it is not surprising the absence of Hfq has a more dramatic effect on *hdeAB* expression than the absence of GcvB.

### 3.8. High Levels of GcvA, GcvB, and Hfq in WT Alters *hdeA*::*lacZ* and *hdeB*::*lacZ* Expression

**Figure 4:**
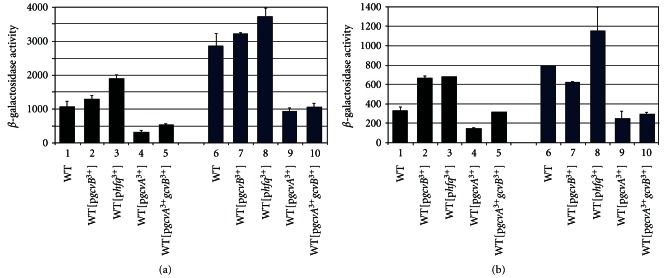
WT*λhdeA*::*lacZ* and WT*λhdeB*::*lacZ* lysogens with the indicated plasmids were grown in LB (black) or GM + glycine (blue) to mid-log phase and assayed for *β*-galactosidase activity.

To verify GcvA negatively regulates and GcvB and Hfq positively regulate the *hdeAB* operon, we transformed WT*λhdeA*::*lacZ,* and WT*λhdeB*::*lacZ* lysogens with plasmids carrying *gcvA*,* gcvB*, both *gcvA *+ *gcvB,* or *hfq*. We hypothesized high GcvB and Hfq would increase expression and high GcvA would repress expression. The lysogens were grown in LB and assayed for *β*-galactosidase. The presence of p*gcvB* ^3+^ resulted in a small increase in *hdeA*::*lacZ* expression and about a 2-fold increase in *hdeB*::*lacZ* (Figures 4(a) and 4(b), lines 1 and 2). The presence of p*hfq* ^3+^ resulted in a 2-fold increase in both *hdeA*::*lacZ* and *hdeB*::*lacZ* expression (Figures 4(a) and 4(b), line 3). The presence of p*gcvA* ^3+^ resulted in a 3.5-fold and a 2.2-fold reduction in *hdeA*::*lacZ* and *hdeB*::*lacZ* expression, respectively (Figures 4(a) and 4(b), line 4). The presence of plasmid p*gcvA* ^3+^ *gcvB* ^3+^ reduced *hdeA*::*lacZ* and *hdeB*::*lacZ* expression, but not to the levels of the p*gcvA* ^3+^ plasmid (Figures 4(a) and 4(b), line 5), suggesting high GcvB antagonize the GcvA effect.

 The lysogens were also grown in GM + glycine. The pattern of regulation was similar to the LB grown lysogens with one exception. The p*gcvB* ^3+^ transformant did not show increased expression of *hdeB*::*lacZ* as in LB (Figure 4(b), compare lines 1 and 2 with lines 6 and 7). It is possible that in WT grown in GM + glycine GcvB is already in excess for regulation. Nevertheless, the results are in agreement with GcvB and Hfq positively regulating the *hdeAB* operon and GcvA negatively regulating the operon.

### 3.9. GcvA, GcvB, and Hfq Regulate *hdeA*::*lacZ* at the Level of Transcription

**Figure 5:**
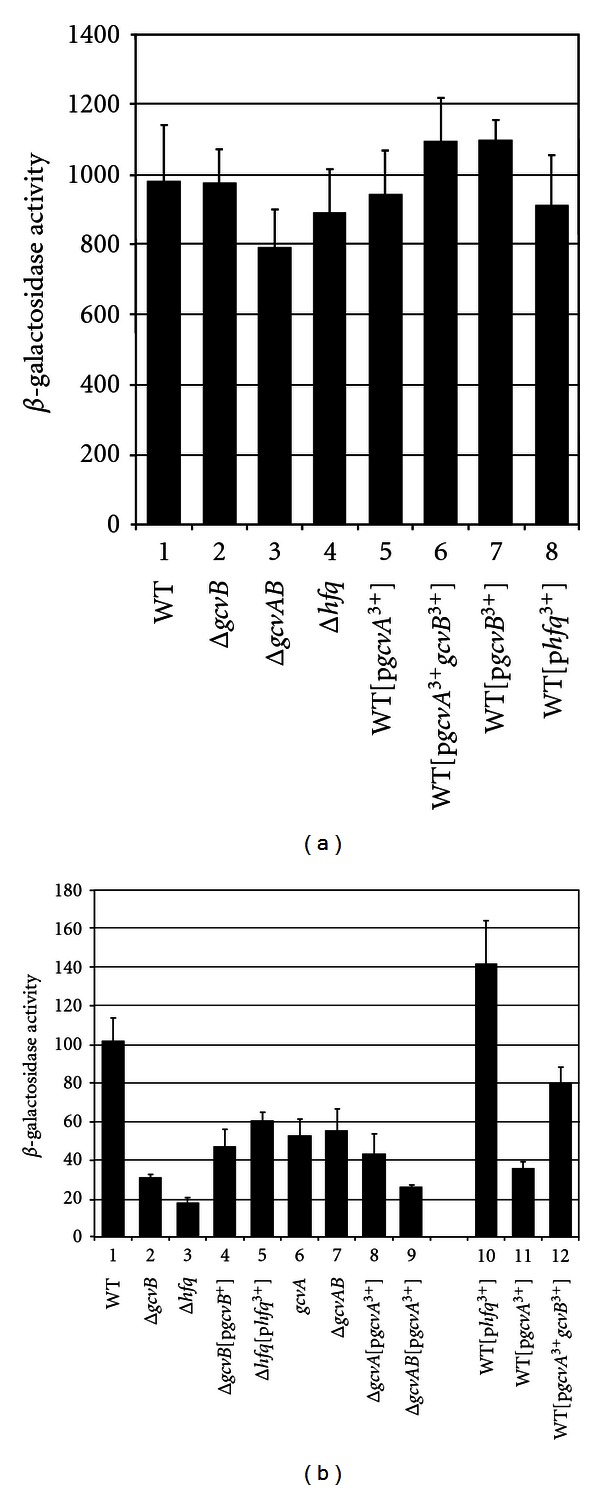
(a) P*_BAD_*::*hdeA*::*lacZ* and (b) *hdeA^−36^*::*lacZ* lysogens were grown in LB to mid-log phase and assayed for *β*-galactosidase activity.

GcvA binds DNA and functions to either activate or repress transcription [27, 35, 48], whereas sRNAs that require Hfq usually regulate posttranscriptionally [19, 21–23]. To determine at what step in regulation of *hdeA* GcvA, GcvB and Hfq function, we constructed a *λ*P*_BAD_*::*hdeA*::*lacZ* fusion where transcription from the P*_BAD_* promoter begins at the +1 start site of the *hdeA* gene (Figure 1(b)). We initially lysogenized a WT strain with the fusion, the lysogen was grown in LB + arabinose (0.0 to 0.2% concentrations) and assayed for *β*-galactosidase. There was a 379-fold induction (2.3 units versus 872 units of activity) at 0.0% and 0.05% arabinose, respectively. This is similar to the level observed from the *λhdeA*::*lacZ* lysogen grown in LB (Figure 3) and confirmed the fusion is inducible by arabinose. We then lysogenized WT, Δ*gcvB*, Δ*gcvAB,* and Δ*hfq* strains. The WT lysogen was also transformed with the plasmids indicated in Figure 5(a). The strains were grown in LB + 0.05% arabinose and assayed for *β*-galactosidase. If GcvA, GcvB, and Hfq regulate at the transcriptional level, we expected they would no longer have an effect on the P*_BAD_*::*hdeA*::*lacZ* fusion. Alternatively, if any of the factors regulates posttranscriptionally, we expected it would still regulate the fusion, as the mRNA is identical to the WT*λhdeA*::*lacZ* mRNA transcript. There was no significant difference in P*_BAD_*::*hdeA*::*lacZ* expression in the WT, WT[p*gcvA* ^3+^] and WT[p*gcvA* ^3+^ *gcvB* ^3+^] transformants (Figure 5(a), compare lane 1 with lanes 5 and 6). The results show GcvA regulates *hdeAB* at the level of transcription. Furthermore, there was no significant difference between WT and the Δ*gcvB*, Δ*gcvAB,* and Δ*hfq* lysogens and the WT[p*gcvB* ^3+^] and WT[p*hfq* ^3+^] transformants (Figure 5(a), compare line 1 with 2, 3, 4, 7 and 8). The results show GcvB and Hfq also regulate at the level of transcription.

 As a complement to the above experiment, we constructed a transcriptional fusion of the *hdeA* promoter 36 bps upstream of the *hdeA* translation start site to a promoterless *lacZYA* operon (Figure 1(a)). Expression of *hdeA* ^−36^::*lacZ* was 3.3- and 5.6-fold higher in WT grown in LB compared to Δ*gcvB* and Δ*hfq* strains (Figure 5(b), lanes 1–3) and activation was partially restored in the Δ*gcvB*[p*gcvB* ^+^] and Δ*hfq*[p*hfq* ^3+^] complemented strains (Figure 5(b), compare lanes 2 and 4 and 3 and 5). The results show GcvB and Hfq still regulate the fusion. Expression in the WT was ~2-fold higher than in the Δ*gcvA* and Δ*gcvAB* lysogens (Figure 5(b), lanes 1, 6 and 7). In the Δ*gcvA*[p*gcvA* ^3+^] and Δ*gcvAB*[p*gcvA* ^3+^] lysogens, there was a 2.4- and 3.9-fold reduction of *hdeA* ^−36^::*lacZ* expression compared to WT (Figure 5(b), compare lanes 1, 8 and 9), showing GcvA negatively regulates the fusion. We also transformed the WT lysogen with plasmids p*hfq* ^3+^, p*gcvA* ^3+^, and p*gcvA* ^3+^ *gcvB* ^3+^. The presence of p*hfq* ^3+^ resulted in a 1.4-fold increase in expression (Figure 5(b), lanes 1 and 10), showing Hfq does positively regulate the fusion. The presence of plasmid p*gcvA* ^3+^ resulted in a 2.7-fold decrease in expression (Figure 5(b), lanes 1 and 11), consistent with GcvA negatively regulating the fusion. The presence of p*gcvA* ^3+^ *gcvB* ^3+^ reduced expression 1.3-fold (Figure 5(b), lanes 1 and 12), suggesting GcvB partially overcome the GcvA effect. The results are consistent with GcvA negatively regulating *hdeAB* at the transcriptional level. Our results also show GcvB and Hfq function during log phase to positively regulate *hdeAB* at the transcriptional level, counterbalancing the negative effect of GcvA on downregulating these genes. GcvB is known to bind Hfq [49]. It is possible GcvB binds to and sequesters Hfq during exponential growth, and the effects observed are due to decreased levels of Hfq to alter regulation of genes such as *rpoS* or the activity of sRNAs such as DsrA and GadY that play roles in acid resistance. Additional studies will verify if GcvA directly binds the *hdeAB* promoter region and how GcvB and Hfq activate the operon.

### 3.10. Effect of pH on *gcvB* Expression

**Figure 6:**
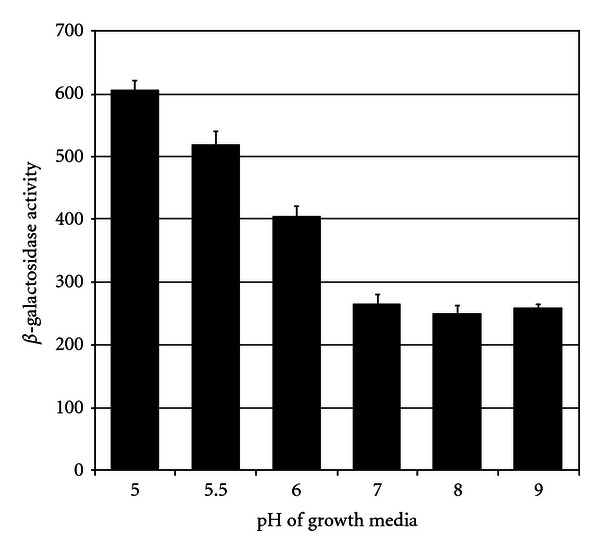
A WT*λgcvB*::*lacZ* lysogen was grown in LB buffered at different pH values to mid-log phase of growth and assayed for *β*-galactosidase activity.

 Our results suggest GcvB plays a role in acid resistance during log phase of growth in rich and minimal media. Therefore, we tested if pH plays a role in regulating *gcvB* expression. A WT*λgcvB*::*lacZ* fusion was grown to mid-log phase in LB at different pH values from 5.0 to 9.0 and assayed for *β*-galactosidase. There was no significant effect from pH 7 to pH 9 on *gcvB*::*lacZ* expression (Figure 6). However, there was a 3-fold increase as the pH was lowered from pH 7 to pH 5 (Figure 6). Since GcvB activates *hdeAB*, an increase in *gcvB* expression at low pH is likely to play a role in final HdeAB levels and in controlling acid resistance.

### 3.11. Effect of GcvA and GcvB on Cell Growth at Low pH

 We carried out studies to show the effects of high GcvA and GcvB levels on growth at low pH. In a Δ*gcvAB* strain transformation with p*gcvA* ^3+^ or p*gcvA* ^3+^ *gcvB* ^3+^ did not significantly alter generation times (GTs) in LB at pH 7 (Table 2). At pH 4.5, GTs of both the WT and Δ*gcvB *strains were significantly increased (Table 2, compare rows 1 and 2, pH 7.0 versus pH 4.5). In addition, in the WT[p*gcvA* ^3+^] strain, with high GcvA and low GcvB, there was a significant increase in the GT compared to the non-transformed WT strain (Table 2, compare rows 1 and 3, pH 4.5). In the *gcvAB*[p*gcvA* ^3+^] transformant, with high GcvA and no GcvB, there was an additional increase in the GT (Table 2, compare rows 3 and 4, pH 4.5 column). In the WT[p*gcvA* ^3+^ *gcvB* ^3+^] and Δ*gcvAB*[p*gcvA* ^3+^ *gcvB* ^3+^] strains, with high GcvA and GcvB, the GTs were not significantly different than in the non-transformed strains (Table 2, compare rows 1 and 2 with rows 5 and 6, pH 4.5). The results are consistent with GcvA negatively regulating acid resistance genes and GcvB overcoming the negative effect of GcvA. The results also show GcvA and GcvB affect acid resistance in log phase cells and could play important roles in the ability of enteric organisms to colonize the GI tract.

### 3.12. Role of GcvB in Cell Physiology

 In *E*. *coli*, GcvB negatively regulates SstT, CycA, OppA, and DppA levels, the serine transporter, glycine transporter and the oligopeptide, and dipeptide periplasmic binding proteins, respectively [19, 22, 23]. These proteins not only transport amino acids and peptides to provide nutrients, but possibly toxins and antibiotics [50, 51]. If conditions that favor relatively high levels of amino acids and small peptides also favor the presence of small toxic compounds, the decreased expression of transport systems for these small molecules by GcvB could prevent transport of toxic compounds into the cell [22]. Our results show that GcvB also positively regulates genes involved in acid resistance. In addition, GcvA, the activator for *gcvB* expression, negatively regulates genes involved in acid resistance. These findings suggest GcvB and GcvA play important roles in the ability of *E*. *coli* to survive low pH conditions. Recently, in a screen of a sRNA gene knockout library, GcvB was shown to enhance *E*. *coli* survival at low pH [25]. Thus, GcvB likely allows *E*. *coli* to respond to and survive two stress conditions, the presence of toxic compounds and low pH environments. Both of these conditions are encountered as *E*. *coli* moves from an external environment into the GI tract. Understanding the biological roles of GcvB and GcvA in acid resistance and their mechanism(s) of regulation will provide insights as to how cells respond to environmental challenges to infect host organisms.

## Figures and Tables

**Table 1:** Strains, plasmids, and phage.

Strain, plasmid, or phage	Relevant genotype	Source
Strain*		
GS162	WT	This lab
GS776	*cycA30*::Tn*10 *	This lab
GS854	*argA81*::Tn*10 *	This lab
GS998	GS162 *gcvA *	[27, 28]
GS1132	GS162 Δ(*gcvAgcvB*)::Ω*aadA* (referred to as Δ*gcvAB*)	[19]
GS1144	GS162 Δ*gcvB*::ΩCM^R^ (referred to as Δ*gcvB*)	[21]
GS1148	GS162 *hfq-1*::ΩCM^R^ (referred to as Δ*hfq*)	[23]
Plasmid		
pGS554	Single-copy vector + constitutive *gcvB* (p*gcvB* ^2+^)	[19]
pGS571	Multicopy vector + WT *gcvB* (p*gcvB* ^3+^)	[29]
pGS594	Single-copy vector + WT *gcvB* (p*gcvB* ^+^)	This lab
pGS609	Multi-copy vector + WT *hfq* (p*hfq * ^3+^)	[23]
pGS611	Multi-copy vector + WT *gcvA* (p*gcvA* ^3+^)	This study
pGS624	Multi-copy vector + WT *gcvA gcvB* (p*gcvA* ^3+^ *gcvB* ^3+^)	This study
Phage		
*λ*gt2	*λ* cloning vector; cI857 repressor	[30]
*λhdeA*::*lacZ *	*λ* vector carrying WT *hdeA*::*lacZ* fusion	This study
*λhdeB*::*lacZ *	*λ* vector carrying WT *hdeB*::*lacZ* fusion	This study
*λhdeA* ^−36^::*lacZ *	*λ* vector carrying *hdeA* ^−36^::*lacZ* transcriptional fusion	This study
*λ*P*_BAD_*::*hdeA*::*lacZ *	*λ* vector carrying *hdeA*::*lacZ* fusion under control of the P*_BAD_* promoter	This study

*All strains also carry the *pheA905 thi araD129 rpsL150 relA1 deoC1 flbB5301 ptsF25 rbsR* mutations.

**Table 2:** Effect of GcvA and GcvB on growth at low pH.

Strain	GT (min) grown in LB at	
	pH 7.0	pH 4.5*
(1) WT	52 ± 5	133 ± 2
(2) Δ*gcvAB *	56 ± 2	160 ± 6
(3) WT[p*gcvA* ^3+^]	67 ± 7	306 ± 9
(4) Δ*gcvAB*[p*gcvA* ^3+^]	60 ± 6	437 ± 45
(5) WT[p*gcvA* ^3+^ *gcvB* ^3+^]	67 ± 7	157 ± 4
(6) Δ*gcvAB*[p*gcvA* ^3+^ *gcvB* ^3+^]	62 ± 1	150 ± 5

*Cultures were tested at the end of the experiment to verify the pH had not changed.
